# Prognostic Analysis of Duodenal Gastrointestinal Stromal Tumors

**DOI:** 10.1155/2018/4812703

**Published:** 2018-02-20

**Authors:** Liwen Hong, Tianyu Zhang, Yun Lin, Rong Fan, Maochen Zhang, Mengmeng Cheng, Xiaolin Zhou, Juntao Sun, Peijun Sun, Qiangqiang Wu, Lei Wang, Zhengting Wang, Jie Zhong

**Affiliations:** ^1^Department of Gastroenterology, Ruijin Hospital, Shanghai Jiaotong University School of Medicine, Shanghai, China; ^2^Department of Cadre Ward, Fujian Medical Union Hospital, Fujian, China

## Abstract

**Aim:**

This study aims to analyze factors possibly related to the prognosis of duodenal gastrointestinal stromal tumors (DGISTs).

**Methods:**

We collected and retrospectively analyzed clinical and pathological data of 62 patients with primary DGISTs. All the patients were hospitalized and received complete surgical resection at Shanghai Ruijin Hospital from September 2003 to April 2015. We followed up the patients to determine survival outcomes. We also analyzed the effect of clinical and pathological factors on disease-free survival (DFS) and overall survival (OS) of the patients.

**Results:**

Kaplan-Meier univariate survival analysis demonstrated that tumor size, mitotic index, Ki-67 index, and pathological risk were correlated with the DFS and OS of the patients (DFS *P* = 0.039, 0.001, <0.001, and 0.005, resp.; OS *P* = 0.027, 0.007, <0.001, and 0.012, resp.). Cox multivariate regression analysis revealed that Ki-67 index was an independent prognostic factor affecting DFS and OS (*P* = 0.007 and 0.028, resp.). Moreover, Kaplan-Meier survival analysis showed that imatinib treatment for patients with recurrence was correlated with prolonged OS (*P* = 0.002).

**Conclusion:**

Prognosis for DGIST treated by R0 resection is favorable. High level of Ki-67 can be an independent risk factor of DGIST prognosis. Adjuvant imatinib therapy for patients with tumor recurrence could probably lead to prolonged survival.

## 1. Introduction

Gastrointestinal stromal tumors (GISTs) are the most common mesenchymal tumors of the gastrointestinal tract. GISTs are characterized by spindle, pleomorphic, or epithelioid tumor cells and are stained positive for CD117 (c-kit), CD34, actin, desmin, S-100, or Ki-67 [1]. Gastric GISTs (60%) and intestinal GISTs (30%) are the most common types, whereas duodenal GISTs (DGISTs) are rare (5%) [1]. The prognosis of GISTs is correlated with tumor site, tumor size, mitotic count, and Ki-67 expression [2, 3]. Despite increasing studies on DGISTs, prognostic analyses remain limited because of the rarity of these tumors. In this retrospective study, we focus on several potential factors, such as clinicopathological parameters and imatinib treatment, to predict disease-free survival (DFS) and overall survival (OS) of 62 patients with DGISTs.

## 2. Materials and Methods

### 2.1. Patients

We recruited 62 patients diagnosed with primary DGISTs and received operative resection at Shanghai Ruijin Hospital from March 2003 to March 2015. All the patients were evaluated according to standard diagnostic criteria. The patients had no metastatic disease or other malignant tumors and did not undergo preoperative chemotherapy or radiotherapy. The patients received complete tumor resection (R0 resection) with negative margins, and the diagnosis of DGIST was confirmed through pathological analysis [1].

### 2.2. Clinical Data

We obtained clinical data from the medical history of the patients. General data, including gender, age, and clinical symptoms (abdominal pain and distention, bloody stool, abdominal mass, and icterus), surgical methods, and postoperative treatment were recorded.

### 2.3. Pathological and Immunochemical Data

Two senior pathologists reviewed all pathological sections to obtain a clear diagnosis. The tumors were mainly composed of spindle cells and few epithelioid cells. Tumor size was defined as the maximum diameter of the surgical tumor specimen. All the DGIST specimens had tumor-negative margin. Mitotic count was determined from 200 adjacent high-power fields (HPFs) within the most active area of karyomitosis. The average mitotic count in 50 HPFs was specified as the mitotic count for the patient. A 5 *μ*m section was cut from the paraffin-embedded tumor tissues. Monoclonal antibody against Ki-67, namely, clone MIB-1 (DAKO, dilution of 1 : 100), was used to cover the slide following the standard step of Envision methodology. The percentage of cells stained with Ki-67 was determined in HPFs with 40x object lens, with at least 500 tumor cells. Pathological risk was graded according to the National Institutes of Health (NIH) consensus risk criteria.

### 2.4. Follow-Up Study

Survival data were obtained by telephone consultation, outpatient service, and hospital imaging until August 2016. We conducted postsurgery follow-up every 3 months for the first year, every 6 months for 3–5 years, and every year after 5 years. Fifty-five of 62 patients were followed up. DFS was defined as the interval from the time when the tumor was completely removed to the time of its recurrence. For patients without tumor recurrence, DFS was defined as the interval from the time when the tumor was completely removed to the time of the latest follow-up. OS was defined as the interval from the time when the tumor was completely removed to the time of death. For patients without records of death, OS was defined as the interval from the time when the tumor was completely removed to the time of the latest follow-up.

### 2.5. Statistical Study

Statistical analysis was performed using SPSS V23.0. Qualitative data were presented by number of cases or ratio of composition. Survival and univariate analyses (gender, age, clinical symptom, tumor growth pattern, tumor site, tumor size, Ki-67 index, and mitotic count activity) were performed using the Kaplan-Meier method and log-rank test. Multivariate analysis of factors affecting survival was conducted using the Cox proportional hazards regression model. The optimal model was determined using stepwise regression. Differences at *P* < 0.05 were considered statically significant.

## 3. Results

### 3.1. Clinicopathological Characteristics

The success rate of the follow-up was 88.7%; seven patients were not followed up due to change in contact information. The study cohort consisted of 26 men and 29 women (sex ratio, 0.9 : 1). As shown in Table 1, the age range of the patients was 27 to 78 years (average, 53.04 ± 10.85 years). 31 patients (56.36%) presented gastrointestinal hemorrhage or anemia, 17 patients (30.91%) presented abdominal pain or distension, 1 patient (1.82%) presented abdominal mass, and 6 patients (10.91%) were asymptomatic when diagnosed. No patient showed a symptom of icterus. DGISTs primarily occurred in the descending duodenum (49.09%), followed by the horizontal duodenum (38.18%), duodenum bulb (10.91%), and ascending duodenum (1.82%). According to operation records, tumors presented exophytic growth in 37 cases (67.3%), intraluminal growth in 10 cases (18.2%), and both intraluminal and exophytic growth in 8 cases (14.5%).

None of the 55 patients with DGISTs revealed metastasis during preoperative examination, and all the patients received complete surgical resection. Among the patients, 17 (30.91%) received Whipple/Child procedure, during which the pancreas was partially removed and 38 (69.09%) received local resection. Tumor size ranged from 1.2 to 10 cm (average size, 4.26 ± 2.23 cm), and tumor diameter was greater than 5 cm in 38 patients (69.09%). We performed a mutation analysis for CD117 and PDGFA. All patients were CD117 positive, and none was PDGFA positive.

### 3.2. DFS and OS Analyses

As of August 31, 2016, the median of the follow-up was 66 months (range, 12–155 months). Postoperative tumor recurrence was found in 11 patients at a median time of 24 months (range, 4–121 months), and 6 of them died of tumor recurrence. None of the patients without tumor recurrence died during the follow-up.

**Figure 1:**
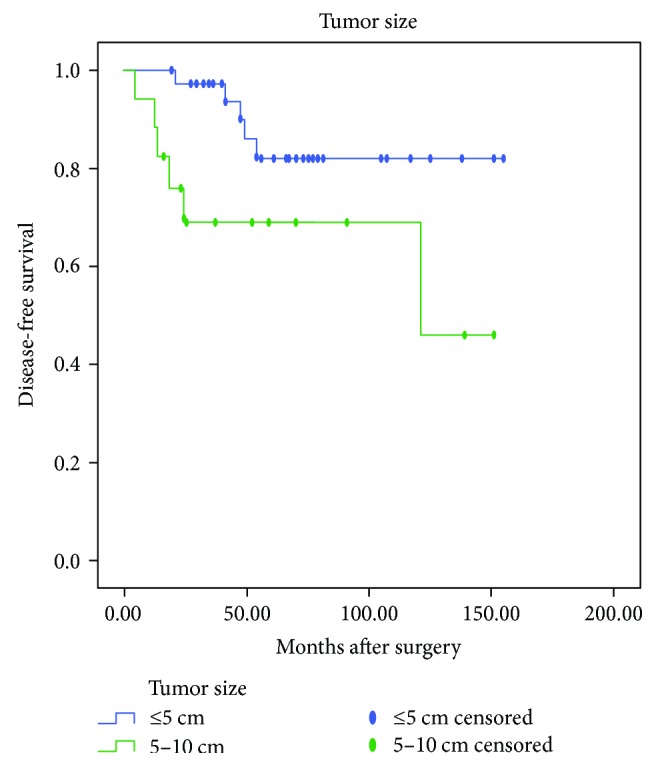
The Kaplan-Meier survival analysis of tumor size > 5 cm predicts a poorer prognosis of duodenal stromal tumor.

**Figure 2:**
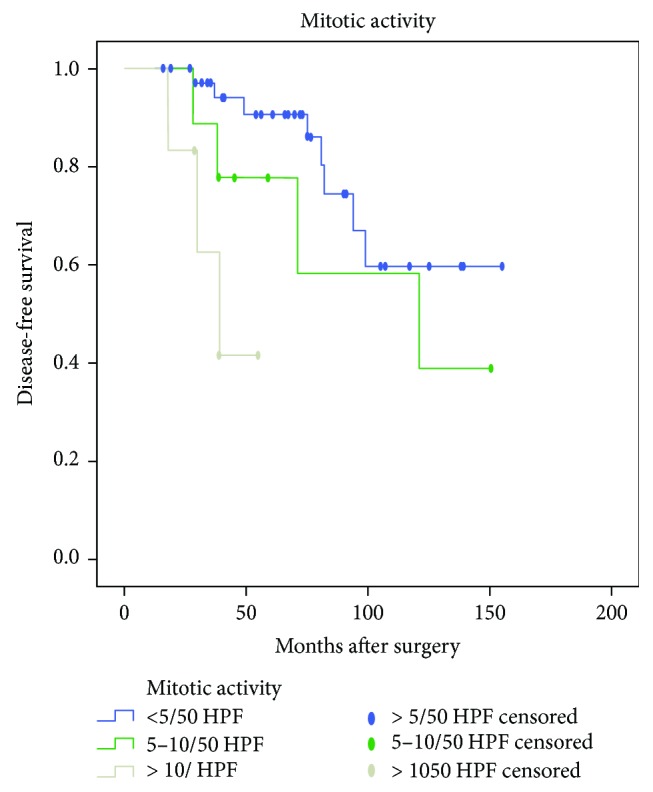
The Kaplan-Meier survival analysis of different levels of mitotic activity indicates the different prognoses of duodenal stromal tumor. Higher mitotic activity predicts a poorer prognosis.

**Figure 3:**
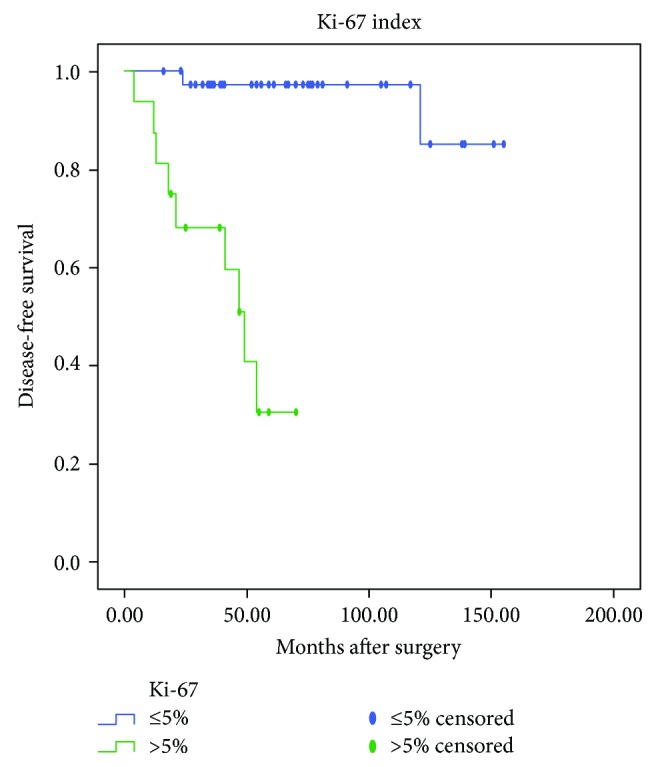
The Kaplan-Meier survival analysis of Ki-67 indicates that index > 5% predicts a poorer prognosis of duodenal stromal tumor.

**Figure 4:**
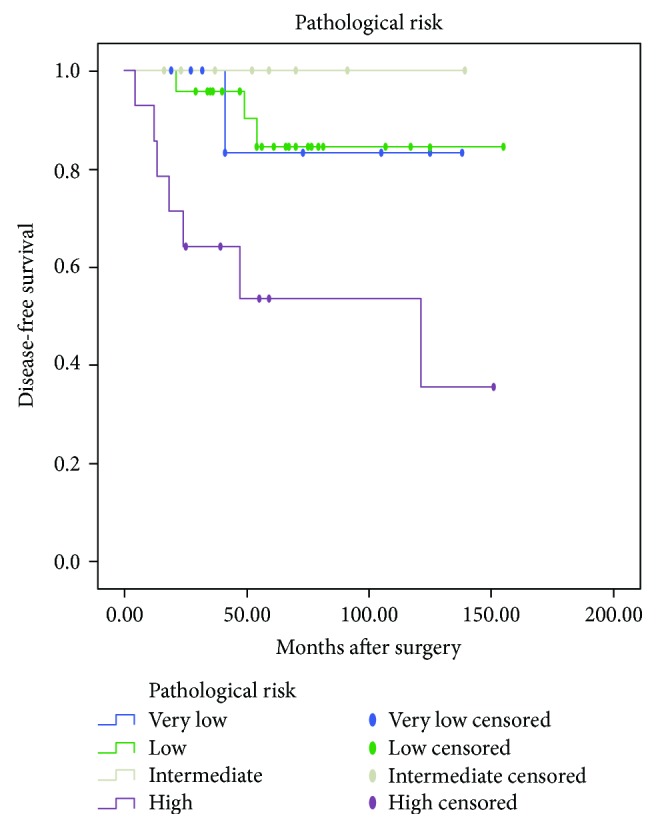
The Kaplan-Meier survival analysis reveals that the lower the pathological risk, the better the prognosis of duodenal stromal tumor.

Univariate analysis using Kaplan-Meier product-limit method was compared by using the log-rank test. DFS calculations were performed separately based on patients' symptoms, age, tumor site, size, and growth pattern, surgical method, mitotic activity, Ki-67 index, pathological risk, and imatinib treatment after surgery and after tumor recurrence. As shown in Table 1, Kaplan-Meier survival analysis demonstrated no statistically significant differences (*P* > 0.05) in DFS or OS between male and female, different age groups (less than 60 years old and over 60 years old), neither between groups of patients with and without symptoms, patients undergone surgery with different methods, tumors with different growth patterns. Differences in DFS and OS between the group with tumor size of less than 5 cm and group with tumor size of more than 5 cm were statistically significant (*P* = 0.039 and 0.027, resp.; Figure 1). The smaller the tumor size, the longer the DFS and OS. However, no difference in DFS or OS was confirmed between the group with tumor size of less than 2 cm and the group with tumor size of 2–5 cm. Differences in DFS and OS among groups with different mitotic counts were statistically significant (*P* = 0.001 and 0.007, resp.; Figure 2). Hence, higher mitotic activity predicts poorer prognosis of DGIST. In addition, differences in DFS and OS among groups with different Ki-67 expressions were also statistically significant (*P* < 0.001 and <0.001, resp.; Figure 3). Lower Ki-67 expression indicates longer DFS and OS. Furthermore, differences in DFS and OS among groups with different levels of pathological risk had statistical significance (*P* = 0.005 and 0.012, resp.; Figure 4), which reveals that lower pathological risk may lead to better DGIST prognosis.

We used Cox multivariate regression analysis to analyze the factors with *P* value below 0.010 (gender, tumor size, mitotic activity, and Ki-67 index) according to the univariate analyses of DFS and OS to better analyze the prognosis of patients with DGIST. Pathological risk was not included in the analysis because it could interfere with the weight coefficient of other factors. Increased Ki-67 expression was an independent risk factor for tumor recurrence and OS (*P* = 0.005 and 0.017, resp.; Table 2).

In this study, we also considered the imatinib adjuvant therapy in patients with DGIST. Imatinib had become the first-line drug therapy for patients with GIST suffering from intermediate-high pathological risk [1]. Therefore, we performed Kaplan-Meier survival analysis of DFS and OS to evaluate the efficacy of imatinib for patients with intermediate-high pathological risk. In this study, 22 patients had intermediate-high pathological risk, and among them, 6 used imatinib after surgery. The *P* values of DFS and OS were 0.489 and 0.804, respectively; these values could not support the preventive use of imatinib in patients with DGIST after R0 resection to prolong survival time and reduce tumor recurrence. Besides, we performed Kaplan-Meier survival analysis of the OS of the postsurgery treatment of imatinib in patients with recurrence. As a result, a *P* value of 0.002 represented a statistical significance and signified that imatinib adjuvant therapy may lead to longer survival time in DGIST patients with tumor recurrence.

## 4. Discussion

GIST prognosis correlates with tumor site, tumor size, and mitotic count according to current research. Large tumor size and high mitotic count are related to poor prognosis [4, 5]. In recent years, DGIST has been increasingly reported. In this study, 55 cases of DGISTs showed similar histological and morphological characteristics of GIST, with positive CD117 expression found via immunohistochemical assay. Male-to-female patient ratio was 0.9 : 1 in this study, with a median onset age of 52 years old. The previous literature has reported that GIST has a fairly equal gender distribution [6]. Other research also reports that the median age at onset is 50–55 years old, which is similar to the findings of this study [7].

DGIST has no specific clinical presentation. In this study, the most frequent clinical symptom is gastrointestinal bleeding, as previously reported [8]. DGISTs are mainly located in the duodenal muscle layer and may grow into the submucosa and lamina propria [9], leading to mucosal ulceration and hemorrhage. Similar to recent reports [10, 11], the main location of DGISTs in this study is the descending of duodenum and followed by the horizontal duodenum.

Curative treatments for GIST consist of surgical resection with negative surgical margins without tumor rupture. The probability of undergoing pancreaticoduodenectomy increased when DGIST was large or located in the descending of duodenum, because the ampulla of Vater and pancreatic head were often involved. However, disagreements exist over the optimal surgical procedures for DGIST [12]. In this study, no surgery-related death was noted. Thus, we assumed that these operations were safe and reliable. We also drew conclusion from survival analysis that the type of surgical procedure did not affect the prognosis of DGIST, which is consistent with existing research [11].

In the current series, the 5-year OS and DFS rates were 75% and 72%, respectively, which were similar to the results of other reports [8, 13]. By contrast, the 5-year DFS rate of GISTs in jejunum and ileum was lower (about 40%) than that of DGISTs [14]. We suggested that favorable prognosis of DGISTs may be related with early clinical symptoms and diagnosis. We find that there may be more symptomatic cases of DGIST than GIST in the stomach and small intestine than with current reports [15, 16], which may lead to earlier diagnosis and treatment and a smaller average tumor size than GISTs in other sites.

Tumor size and site and mitotic count are the classical indicators used to estimate the biological characteristics of GIST. The average tumor size of DGISTs was smaller than GISTs in other sites reported in the current literature [17]. Previous studies have suggested that tumor size is the only important independent prognostic factor in multivariate analysis for DFS after R0 resection of gastric GISTs [18], while others argue that the mitotic index was an independent factor in multivariate analysis [19, 20]. DeMatteo et al. found through multivariate analysis that mitotic rate ≥ 5, tumor size ≥ 10 cm, and primary tumor location were independent factors for recurrence [21]. In the present study, tumor size, mitotic activity index, Ki-67 index, and pathological risk are prognostic factors for DGIST according to Kaplan-Meier analysis, in which Ki-67 index tends to be the most important factor according to Cox regression (*P* = 0.017). Ki-67 is a type of nucleoprotein expressed in all phases of cell proliferation, except for the stationary phase. Thus, Ki-67 detection can be applied for assessment of DNA ploidy and telomerase activity [22]. High levels of Ki-67 may demonstrate early oncological recurrence. However, the reliability of tumor size is uncertain, partly because the number of patients included is limited. We did not have patients with tumor size > 10 cm, which had been reported in other studies with higher postsurgical recurrence rate. Different study end points may give different tumor sizes: study end points were DFS rate in some present studies and OS rate in others. Secondly, mitotic count is still controversial because mitosis identification can be subjective, and the number of detected cases may vary. Differences in surgeon's personal performance and surgical procedures may also lead to different observational results in tumor prognosis. In addition, biases could also arise from the low incidence of DGIST and small sample scale among individual studies. Further studies on this topic are required to confirm our results.

Some reports demonstrated that a combination of surgery and targeted therapy may reduce the recurrence rate or slow down the disease progression [23]. Imatinib, a type of tyrosine inhibitor antagonistic to c-kit, PDGFR, and ABL kinase, is the first-line treatment of metastatic or unresectable GISTs. Randomized phase III clinical trials assessing the role of imatinib adjuvant therapy proved that it could prolong DFS for patients with R0 resected, high-risk GISTs [24]. Preventive use of imatinib in patients after R0 resection of DGISTs of intermediate-high pathological risk was not proven. However, imatinib adjuvant therapy in patients with tumor recurrence could provide a longer survival time.

Small sample size limits the present study. Small-scale survival analyses may mislead the results. Therefore, we should be cautious in evaluating results. In the future, prospective studies including larger numbers of DGIST patients will be needed.

## 5. Conclusion

In summary, prognosis for DGIST treated by R0 resection is favorable. In this study, high level of Ki-67 could be an independent risk factor of DGIST prognosis. Adjuvant imatinib therapy for patients with tumor recurrence could probably lead to prolonged survival.

## Figures and Tables

**Table 1:** Clinical and pathological features for 55 cases of DGIST associate with disease-free survival (DFS) and overall-survival (OS).

Characteristic	*N* (%)	DFS (month)	Chi-square value	*P* value	OS (month)	Chi-square value	*P* value
Gender			3.383	0.066		3.451	0.063
Male	26 (47.27)	101.7 ± 10.91			122.5 ± 11.10		
Female	29 (52.73)	137.4 ± 9.292			147.3 ± 7.249		
Age			0.584	0.445		0.047	0.829
≥60	15 (27.27)	132.2 ± 12.26			130.3 ± 12.91		
<60	40 (72.73)	120.8 ± 9.744			138.3 ± 7.716		
Symptoms			0.01	0.921		0.35	0.554
Asymptomatic	6 (10.91)						
Abdominal pain/distention	17 (30.91)						
Hemorrhage/anemia	31 (56.36)						
Abdominal mass	1 (1.82)						
Surgical method			2.16	0.142		1.308	0.253
Whipple/child	18 (32.73)	63.30 ± 6.771			73.58 ± 4.941		
Local resection	37 (67.27)	131.6 ± 8.522			140.0 ± 6.8		
Growth pattern			2.637	0.268		0.62	0.733
Exophytic growth	37 (67.3)	121.7 ± 7.185			123.2 ± 8.342		
Intraluminal growth	10 (18.2)	94.14 ± 19.40			144.0 ± 8.981		
Both	8 (14.5)	116.8 ± 21.05			117.8 ± 20.39		
Tumor size			4.281	0.039		4.867	0.027
≤5 cm	17 (30.91)	135.1 ± 8.105			144.4 ± 6.695		
>5 cm	38 (69.09)	101.9 ± 15.67			112.7 ± 16.32		
Tumor size			0.003	0.958		0.039	0.844
≤2 cm	9 (16.36)	121.8 ± 14.76			132.7 ± 4.355		
2~5 cm	29 (52.73)	135.4 ± 8.946			146.1 ± 8.200		
Site			2.548	0.467		2.496	0.476
Bulb	6 (10.91)						
Descending	27 (49.09)						
Horizontal	21 (38.18)						
Ascending	1 (1.818)						
Mitotic activity			14.25	0.001		10.05	0.007
<5/50 HPF	40 (72.73)	123.3 ± 9.080			144.4 ± 6.695		
5~10/50 HPF	9 (16.36)	103.4 ± 17.88			121.5 ± 18.29		
>10/50 HPF	6 (10.91)	40.29 ± 6.047			59.60 ± 10.65		
Ki-67 index			26.21	<0.001		12.92	<0.001
≤5%	38 (69.09)	147.3 ± 5.136			151.5 ± 3.492		
>5%	17 (30.91)	43.88 ± 6.000			92.53 ± 12.63		
Pathological risk			25.28	0.005		11	0.012
Very low	9 (16.36)						
Low	24 (43.64)						
Intermediate	9 (16.36)						
High	13 (23.64)						
Imatinib after surgery for middle-high pathological risk			0.478	0.489		0.062	0.804
Yes	6 (27.27)	41.17 ± 7.531			58.00 ± 11.43		
No	16 (72.73)	111.3 ± 14.79			122.7 ± 14.55		
Imatinib after recurrence						9.811	0.002
Yes	6 (54.55)				125.6 ± 12.16		
No	5 (45.45)				35.73 ± 12.71		

**Table 2:** Multivariate analysis of predictive factors for recurrence.

Factors	Odds ratio for DFS (95% CI)	*P* value for DFS	Odds ratio for OS (95% CI)	*P* value for OS
Gender	0.626 (0.133-2.950)	0.554	0.391 (0.033-4.694)	0.459
Tumor size (≤5 versus >5 cm)	1.672 (0.394-7.086)	0.486	1.936 (0.687-69.95)	0.144
Mitotic activity (<5 versus 5~10 versus >10/50 HPF)	1.722 (0.754-3.931)	0.197	0.561 (0.443-6.926)	0.434
Ki-67 index (≤5% versus >5%)	19.714 (2.243-173.2)	0.007	3.442 (0.002-0.537)	0.028
